# Projecting the Potential Global Distribution of Sweetgum Inscriber, *Acanthotomicus suncei* (Coleoptera: Curculionidae: Scolytinae) Concerning the Host *Liquidambar styraciflua* Under Climate Change Scenarios

**DOI:** 10.3390/insects15110897

**Published:** 2024-11-18

**Authors:** Kaitong Xiao, Lei Ling, Ruixiong Deng, Beibei Huang, Yu Cao, Qiang Wu, Hang Ning, Hui Chen

**Affiliations:** 1 Hubei Key Laboratory of Biological Resources Protection and Utilization, Hubei Minzu University, Enshi 445000, China; 13402709191@163.com (K.X.); marsyu0722@163.com (R.D.); hbei0825@163.com (B.H.); zhedaotinihui@163.com (Y.C.); lxwq20030226@163.com (Q.W.); 2 College of Forestry and Horticulture, Hubei Minzu University, Enshi 445000, China; 3 College of Biology, Hunan University, Changsha 410082, China; linglei@hnu.edu.cn; 4 State Key Laboratory for Conservation and Utilization of Subtropical Agro-Bioresources, Guangdong Key Laboratory for Innovative Development and Utilization of Forest Plant Germplasm, College of Forestry and Landscape Architecture, South China Agricultural University, Guangzhou 510642, China

**Keywords:** *Acanthotomicus suncei*, climate change, species distribution models, potential distribution, pest management

## Abstract

*Acanthotomicus suncei* is a newly discovered bark beetle in China that significantly threatens the American sweetgum *Liquidambar styraciflua*. In recent years, this pest has spread from its original habitat to many surrounding cities, causing substantial economic and ecological losses. Considering the wide global distribution of its host, *Liquidambar styraciflua*, this pest is likely to continue to spread and expand. Once the pest colonizes a new climatically suitable area, the consequences could be severe. Therefore, we employed the CLIMEX and Random Forests model to predict the potential suitable distribution of *A. suncei* globally. The results showed that *A. suncei* was mainly distributed in Southern China, in South Hokkaido in Japan, Southern USA, the La Plata Plain in South America, southeastern Australia, and the northern Mediterranean; these areas are located in subtropical monsoon, monsoonal humid climates, or Mediterranean climate zones. Seasonal rainfall, especially in winter, is a key environmental factor that affects the suitable distribution of *A. suncei*. Under future climates, the total suitable area of *A. suncei* is projected to decrease to a certain extent. However, changes in its original habitat require serious attention. We found that *A. suncei* exhibited a spreading trend in Southwest, Central, and Northeast China. Suitable areas in some countries in Southeast and South Asia bordering China are also expected to show an increased distribution. The outward spread of this pest via sea transportation cannot be ignored. Hence, quarantine efforts should be concentrated in high-suitability regions determined in this study to protect against the occurrence of hosts that may contain *A. suncei*, thereby avoiding its long-distance spread. Long-term sentinel surveillance and control measures should be carried out as soon as *A. suncei* is detected, especially in regions with high suitability. Thus, our findings establish a theoretical foundation for quarantine and control measures targeting *A. suncei*.

## Simple Summary

*Acanthotomicus suncei* is a newly discovered bark beetle in China that causes substantial mortality to American sweetgum *Liquidambar styraciflua* individuals. Currently, the number of host trees killed by this bark beetle is increasing. Climate change has exacerbated this problem, causing its range to expand. Considering the wide global distribution of its host, *Liquidambar styraciflua*, the pest is likely to continue to spread and expand. Here, using CLIMEX and the Random Forests model, we projected the potential global distribution of *A. suncei* concerning the host *L. styraciflua*. Under future change scenarios, the total suitable area is projected to decrease to a certain extent. However, the changes in its original habitats provide three spreading trends to Southwest, Central, and Northeast China. Meanwhile, suitable areas in some countries in Southeast and South Asia bordering China are also expected to show an increased distribution, which poses substantial challenges for forest managers. Our projected results provide significant data regarding the current and future potential distribution of *A. suncei* worldwide, which could be used as a reference for recognizing areas currently vulnerable to potential invasions by this pest.

## 1. Introduction

*Liquidambar styraciflua* L., the American sweetgum, is indigenous to North and Central America and is extensively found throughout the southeastern USA, from Connecticut to central Florida and eastern Texas, westward to Missouri, Arkansas, and Oklahoma, and northward to southern Illinois [1]. This deciduous tree belongs to the subtropical humid climate tree species, which is often used as an ornamental tree in street and landscape architecture, benefiting from its rounded crown, upright stem, and colorful foliage in autumn landscape characteristics [2]. Currently, *L. styraciflua* is broadly cultivated in temperate areas around the world. In 1681, John Banister brought *L. styraciflua* to Europe for the first time and planted them in the gardens of Fulham Abbey [3]. Henceforth, *L. styraciflua* quickly spread to other regions in Europe as a prayer tree. *L. styraciflua* is very popular locally, primarily because of its versatile leaf colors in autumn, including green, orange, red, and bright purple [4]. Hence, many European cities have also started planting it extensively as a major ornamental tree. In Asia, the cultivation history of *L. styraciflua* in China indicates that it was introduced to China as a material for extracting balm as early as 1956 [5]. Since about 2000, *L. styraciflua* has been widely planted and promoted as an ornamental tree with the continuous urbanization of China [6]. At present, *L. styraciflua* has been widely planted in East China, specifically in Shanghai City and Shandong, Jiangsu, Zhejiang, Anhui, Fujian, Jiangxi, and Shandong Provinces [7]. The existence of *L. styraciflua* in temperate regions is the best historical witness in the history of global horticultural cultivation and urbanization. However, the emergence of a newly discovered pest, *Acanthotomicus suncei* Gao & Cognato (Coleoptera: Curculionidae, Scolytinae), in China may threaten the future of *L. styraciflua*.

In 2013, a borer pest emerged in the Yangtze River Delta of China, causing a serious hazard to *L. styraciflua*. Expert identification revealed it to be a new pest, *A. suncei* [8]. The earliest record of *A. suncei* can be traced back to 1959 when it caused serious damage to *L. styraciflua* in the Yuhuatai Martyrs, Nanjing City, Jiangsu Province, according to relevant records [6,9]. *A. suncei* is an oligophagous insect, and its original host plant is *Liquidambar formosana* Hance. Compared to the original host, *A. suncei* has a stronger selectivity compared to *L. styraciflua* [6]. Its population exhibited a higher population fitness after the bark beetle fed on *L. styraciflua* [10]. Therefore, the *A. suncei* population outbreak in *L. styraciflua* has been more severe in recent years. The life history of *A. suncei* is mostly hidden inside the bark of *L. styraciflua*, where its reproduction and diffusion speed are fast [11]. Generally, a single tree damaged by *A. suncei* is likely to damage entire *L. styraciflua* forests. Consequently, *A. suncei* is spreading at an unprecedented speed in the Yangtze River Delta, causing the deaths of numerous *L. styraciflua* that were planted in multiple nurseries, urban parks, or green spaces in cities [9].

As a lethal and elusive killer, the threat posed by *A. suncei* to *L. styraciflua* is more serious. First, the beetle usually causes the host to rapidly die within one to two months after invasion [6]. The hazard symptoms are often subtle or nonexistent, posing huge challenges in identifying and confirming the infestation [12]. This delays the implementation of subsequent pest controls. According to statistics, *A. suncei* killed more than 30,000 *L. styraciflua* in Shanghai City and Jiangsu Province from 2013 to 2018, causing huge ecological and economic losses [6]. Most dead trees are found in nurseries; hence, apart from the natural dispersion of *A. suncei*, the trade and transportation of *L. styraciflua* seedlings may be an important route for its long-distance spread. Susaeta et al. estimated that the potential economic losses to USA plantation owners could be over USD 150 million if *A. suncei* crossed the ocean and arrived in *L. styraciflua* forests in North America [13]. Li et al. estimated that the potential economic losses of the nursery stock and urban areas could range from USD 12.81 to 14.41 million if *A. suncei* were to become established in the main *L. styraciflua* business [12]. Field prevention and control experiments have indicated that many prevention and control measures cannot effectively control the occurrence and spread of *A. suncei* [6]. Currently, the direct destruction of infected hosts may be the only effective method to control the spread of *A. suncei*. Judging from the current dire situation, further expansion of the infestation is inevitable; however, the specific areas of *A. suncei* dispersal remain unknown. Projecting the potential distribution areas of the bark beetle and further speculating on its possible future path of propagation can facilitate prioritizing surveillance in advance, thereby minimizing losses.

Species distribution models (SDMs), which are often referred to as ecological niche models or habitat suitability models, combine species distribution data with environmental layers to estimate the potential ecological niches of targeted species according to specific algorithms, then project this relationship into a geographic space to indicate the extent to which species favor particular ecological environments [14]. As important prediction tools in ecology, SDMs are widely applied to evaluate the spreading risk of invasive species, plan and manage the habitat ranges of animals and plants, and project the impact of past and future climate change on species and communities. SDMs can be categorized into correlative and mechanistic models [15]. Correlative models, such as typical BIOCLIM, DOMAIN, ENFA, GARP, and machine learning algorithms, use correlation to establish corresponding relationships between species distribution data and environmental variables, matching species distribution with environmental conditions for modeling [16,17]. Hence, correlation models provide important insights into understanding the relationship between species distributions and survival environments [16]. Mechanism models, also known as physiological–ecological or process models, such as popular CLIMEX, mainly rely on the physiological–ecological needs of targeted species and then model this based on species physiological data [18,19]. Therefore, mechanistic models can better explain the biological significance of species survival. However, the modeling accuracy of a single SDM has limitations. Recently, many studies have demonstrated that combining multiple SDMs to predict species distribution can improve prediction accuracy, which has become a trend when using SDMs [20,21,22].

The death event of American sweetgum caused by *A. suncei* is not only due to the correlation between its occurrence data and environmental variables, but more importantly, the process of host tree death is caused by pest physiological parameters. Although MAXENT has been used to predict the potential distribution of *A. suncei*, we found that omitting an important variable, the potential distribution of *L. styraciflua*, made the predictions relatively conservative and did not truly reflect the climatic ecological niche of the bark beetle [12]. Therefore, to improve the accuracy of predictions in reality, in our research, CLIMEX was used to predict the distribution of *L. styraciflua* first, then Random Forests (RFs) were used to predict the distribution of *A. suncei*, and finally obtain the potential distribution of *A. suncei* concerning the host *L. styraciflua*.

## 2. Materials and Methods

### 2.1. Acquiring and Processing Distribution Coordinates of A. suncei and L. styraciflua

**Figure 1:**
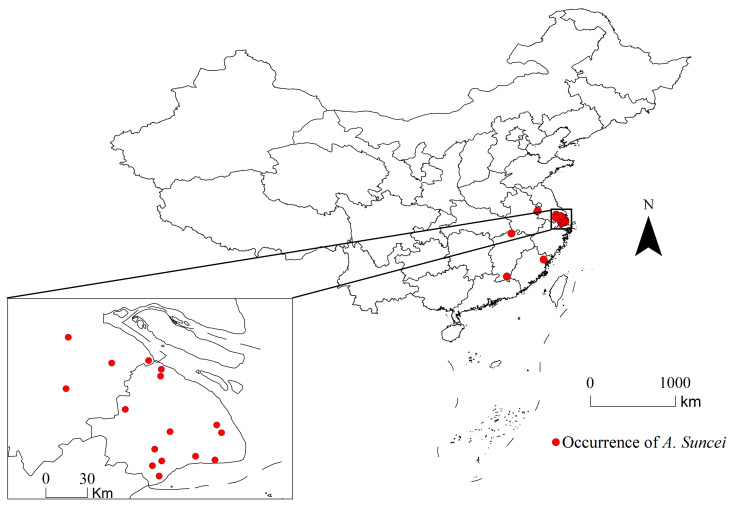
Occurrence coordinates of *A. suncei* in China.

The coordinates of *A. suncei* occurrences were gathered using field surveys and the related literature. From 2021 to 2023, occurrence records of *A. suncei* in the field were collected with assistance from the Department of Forestry Protection. All points, including location name, longitude, latitude, and altitude, were documented. The relevant literature, both domestic and international, was searched for presence records of *A. suncei* [9,12,23,24,25]. In China, occurrence data were collected from the China National Knowledge Infrastructure (CNKI, https://www.cnki.net/) (accessed on 16 March 2024) and the Wanfang Database (https://www.wanfangdata.com.cn) (accessed on 16 March 2024). Coordinates with complete latitudes and longitudes were chosen directly, whereas, for the remaining incomplete locations where only names were provided, the latitudes and longitudes were obtained using Google Maps. Three valid distribution points were obtained from the nine literature pieces. Its scientific name was uploaded to the Web of Science (WOS, https://clarivate.com.cn) (accessed on 16 March 2024) to search for related literature. The occurrence data with incomplete geographic information and minor differences in geographic coordinates were deleted. Finally, 20 occurrence coordinates of *A. suncei* were obtained and are shown in Figure 1 and Table S1.

**Figure 2:**
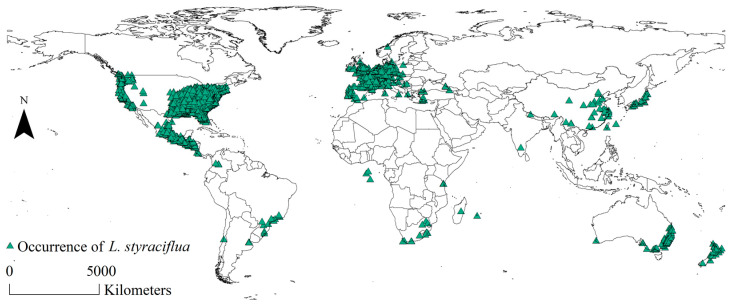
Global presence coordinates of *L. styraciflua*.

For host *L. styraciflua*, 67,996 global distribution coordinates were acquired from the Global Biodiversity Information Facility (GBIF, https://www.gbif.org/) (accessed on 18 March 2024) database (Table S2) [26]. The number of coordinates in China was found to be lower than the actual situation. Therefore, the dataset was further supplemented with 41 *L. styraciflua* distribution coordinates in China and retrieved from the Chinese Plant and Species Information System (iPlant, https://www.iplant.cn/) (accessed on 18 March 2024). Field surveys were also conducted on host distribution. Similarly, some occurrence data with incomplete geographic information and minor differences in geographic coordinates were abandoned. Subsequently, these datasets were merged, and the “Spatially Rarefy Occurrence Data” function within the SDM toolbox of ArcGIS 10.8 (Environmental System Research Institute Inc., Redlands, CA, USA) was utilized to filter the data [27]. The buffer radius was set to 10 km, and 6200 coordinates were obtained for *L. styraciflua*, which are summarized in Figure 2.

### 2.2. Overall Modeling Workflow

Initially, the RF algorithm and CLIMEX 4.0.2 (Hearne Scientific Software, Melbourne, Australia) were applied to forecast the potential distribution of *A. suncei* and its host, *L. styraciflua*, respectively. Considering that studies on host physiology are abundant and sufficient, CLIMEX was selected to predict the potential distribution of *L. styraciflua*. For research on *A. suncei*, 14 studies on this pest were available for reference, including 9 in Chinese and 5 papers from other countries. These studies mainly focus on pest identification, current occurrence status, artificial rearing, and control measures. Research on the physiological aspects of this pest has not been reported. Therefore, realizing the potential distribution of *A. suncei* based on CLIMEX is extremely limited. Considering these limiting factors, RF was selected to project the potential distribution of *A. suncei*. Finally, the RF and CLIMEX prediction result layers were overlayed. Areas with potential distribution of both *A. suncei* and *L. styraciflua* were selected to represent the potential distribution area of *A. suncei* concerning the host [22].

### 2.3. Model Selection and Climate Data Acquisition

CLIMEX utilizes location (.loc) and meteorology (.met) fixed format files, which store the geographic information and climate data of a station, which are combined into a MetManager (.mm) file to be used in the software, respectively [19,28]. The .met files contain data for daily minimum temperature (Tmin), daily maximum temperature (Tmax), monthly precipitation (Rainfall), and relative humidity at 9:00 (RH 0900) and 15:00 (RH 1500). CliMond (http://www.climond.org/) (accessed on 19 March 2024) is a global climatology for bioclimatic modeling, consisting of gridded current climate data and some future climate scenario layers with 10′ or 30′ spatial resolution, on which the standard format for CLIMEX modeling can be downloaded [28]. In this study, the 10′ climate scenario layers were selected for more accurate results. The current meteorological data were sourced from the Worldclim and Climate Research Unit (CRU) (CL1.0 and CL2.0) datasets [29]. The future meteorological data were sourced from global climate model (GCM, also known as the general circulation model) data announced in the IPCC Fourth Assessment Report (AR4) [28]. The IPCC AR4 has four emission scenarios: A1, A2, B1, and B2 [30]. The A1 storyline and scenario family describe a future world with rapid economic growth, a global population that peaks in the mid-century and declines thereafter, and new and more efficient technologies that are rapidly being introduced [20]. The A1 scenario family develops into three groups that describe alternative directions of technological change in the energy system. The three A1 groups are distinguished by their technological emphasis: fossil energy intensive (A1FI), non-fossil energy sources (A1T), and a balance across all sources (A1B) [30]. For this study, the medium greenhouse gas emissions scenario, A1B, was selected to predict the suitable climatic areas of *L. styraciflua*. In general, 30 years is the standard period used to analyze the climate. Hence, the succeeding 30-year period (2030–2060) was selected to project the potential distribution of the host.

For RF modeling, the required bioclimatic layers can be freely accessed using WorldClim (https://www.worldclim.org/). A total of 19 bioclimatic layers with a resolution of 0.5′, 2.5′, 5′, or 10′ were available for download. To match the resolution of CLIMEX modeling, the 10′ bioclimatic layers were chosen in this study. Current climate data were obtained using the thin plate smoothing spline function interpolation method based on the data observed from meteorological stations worldwide [31]. The 2050s were chosen as the future period for forecasting because of the short generation cycle, fast reproduction, and strong adaptability of insects. The IPCC Fifth Assessment Report (AR5) employed representative concentration pathways (RCPs), including RCP2.6 (strict mitigation), RCP4.5 and RCP6.0 (moderate emissions), and RCP8.5 (high emissions) to indicate scenarios where total radiative forcing in 2100 would reach 2.6 W/m^2^, 4.5 W/m^2^, 6 W/m^2^, and 8.5 W/m^2^ above 1750 levels, respectively [32]. Future climate scenarios were generated using downscaled and calibrated global climate models, which were processed by WorldClim [31]. Here, to be essentially consistent with the A1B climate scenario, RCP6.0 was selected to project the future distributions of *A. suncei*.

### 2.4. Using CLIMEX to Project the Potential Distribution of L. styraciflua

#### 2.4.1. CLIMEX Model

CLIMEX is a dynamic simulation model that can be used to project the potential distribution of targeted species. CLIMEX uses the Annual Growth Index (${\mathrm{GI}}_{A}$), which reflects the potential for population growth under suitable growth conditions, and the Stress Index (SI), which describes the cumulative effects of stress during the inclement season, to derive an Ecoclimatic Index (EI) [19]. The EI describes the potential for population growth, with the annual stresses that limit survival and any other limiting factors. The EI scale spans from 0 to 100, with values near 0 indicating that a location is unsuitable as a long-term habitat for a species, while a value of 100 represents optimal habitat conditions [33]. The calculation formula is as follows:

$$\mathrm{EI}={\mathrm{GI}}_{A}\times \mathrm{SI}\times \mathrm{SX}$$

SX, the Stress Interaction Index, is typically disregarded. ${\mathrm{GI}}_{A}$ is primarily affected by the Temperature Index (TI) and the Moisture Index (MI). The calculation formula is as follows:

$${\mathrm{GI}}_{A}=\mathrm{TI}\times \mathrm{MI}$$

SI includes cold stress (CS), heat stress (HS), dry stress (DS), and wet stress (WS), expressed as follows:

$$\mathrm{SI}=\left(1-\frac{\mathrm{CS}}{100}\right)\times \left(1-\frac{\mathrm{HS}}{100}\right)\times \left(1-\frac{\mathrm{DS}}{100}\right)\times \left(1-\frac{\mathrm{WS}}{100}\right)$$

SI was derived through a semi-automated parameter-fitting procedure run in CLIMEX, followed by manual adjustments. The semi-automated parameter-fitting procedure, based on a genetic algorithm, assists users in fitting stress parameters by defining the geographical range where a species experiences minimal stress. This method requires the creation of a reference file based on known species distributions and the setting of appropriate parameter ranges for fitting. Initial species parameter values are needed to start the process, which can be sourced from a species with a similar distribution or from the templates provided by CLIMEX. For more information on CLIMEX, see Kriticos et al. [19].

#### 2.4.2. Parameters Fitting

Specific parameters were set according to the following methods. First, suitable templates were selected, and the initial parameter values were set based on the climate type of the native region (North America) and biological data. Then, 75% of the occurrence records were randomly selected from the total global occurrence records as the training set, and this set was treated as the adjusting basis. Parameters were adjusted to ensure that the selected occurrence records were located in the projected potential distribution of the pest. After the parameters were defined, the remaining occurrence records were used to test the model [34].

**Temperature Index:** Several cultivation experiments indicated that the suitable temperature range for the growth of *L. styraciflua* in original regions was 16–32 °C [35,36,37]. In Nanjing City, China, 15–25 °C was considered a suitable range for *L. styraciflua* growth [5]. Therefore, DV1 and DV2 were set at 16 °C and 32 °C, respectively. The Growth Index (GI) serves as an indicator of population growth potential during a suitable growth period. It generally exhibits higher values during the spring. DV0 can be chosen as a temperature close to early spring. *L. styraciflua* is considered to initiate development at temperatures between 10 and 15 °C [5]. Therefore, DV0 was set at 10 °C. At the southernmost area of *L. styraciflua* cultivation in the USA, the highest temperature could reach 38 °C [1]. DV4 was set at 38 °C.

**Moisture Index:** The lower soil moisture level (SM0) was set at 0.1, which was equivalent to a permanent wilting point for plants with moderate rooting depth [38]. Considering that it is not drought-resistant, the SM0 for *L. styraciflua* was set to 0.1 [1]. The cultivation of *L. styraciflua* seedlings showed that a soil moisture content between 50 and 75% was optimal for seedling growth [39]. Consequently, SM1 and SM2 were set to 0.5 and 0.75, respectively. Along the eastern border of the Mississippi River, *L. styraciflua* is occasionally dominant on the loessial soils of the alluvial flood plain [40]. It is characteristically dominant on the relatively impervious Alfisols of the Illinoian Till Plain, including the poorly drained Avonburg, Blanchester, and Clermont silt loams. Therefore, SM3 was set to 1.8, considering its distribution in alluvial flood plains.

**Cold stress:** Williams and McMillan found that the lowest temperature in the survival area of *L. styraciflua* was −28.9 °C [41]. Therefore, the temperature threshold for cold stress and the cold stress accumulation rate were set to −28.9 °C and 0.9 for Week^−1^, respectively, to fit the distribution area of *L. styraciflua.* Hence, the fitted areas could cover the presence of this species areas in the northernmost USA and Europe.

**Hot stress:** According to the known global distribution of *L. styraciflua* and related records in the literature, the number and the range of distribution of *L. styraciflua* in temperate regions are greater than those in tropical regions. To reflect this difference, the temperature threshold for the heat stress and heat stress accumulation rate were set to 40 °C and 0.2 on Week^−1^, respectively.

**Dry stress:** The soil moisture threshold for dry stress was aligned with SM3, and the dry stress accumulation rate was set at 0.001 week^−1^. To obtain a more accurate fit layer of *L. styracifluas’* presence, the EI values in the dry areas in Central Asia, West Asia, and South Asia and the tropical desert climate regions of Africa were reduced as much as possible without affecting some distribution areas in the climatic arid regions and China.

**Wet stress** Kormanik found that *L. styraciflua* can grow well in areas with high humidity [1]. Therefore, the soil moisture threshold for wet stress and the wet stress accumulation rate were set to 1.8 and 0.001 on Week^−1^, allowing it to exhibit more accurate overlap in areas with higher humidity.

**Hot–wet stress:** To limit the distribution of *L. styraciflua* in wet tropics without affecting its distribution in other regions, the hot–wet stress was set to 28 °C, the hot–wet moisture threshold was set to 1.8, and hot–wet stress rate was set to 0.05 on Week^−1^. Fitting revealed that the fitted range could overlap with the presence areas for *L. styraciflua.*

All the fitted parameter values are summarized in Table 1.

#### 2.4.3. Classification of EI Values

The EI value offers a comprehensive measure of the suitability of a species to particular locations [42]. EI values are typically categorized to assess climate favorability for the species, with classification standards based on the known distribution of the species [43]. An EI value of zero indicates that the location is unfavorable for the long-term survival of the targeted species, which can be used as a dividing cutoff between unfavorable and marginally favorable habitats [42]. Based on the distributional characteristics of *L. styraciflua* in the USA and China, the EI thresholds between marginal and favorable habitats and between favorable and highly favorable habitats can be clearly delineated. In the USA, *L. styraciflua* is primarily found in the eastern regions, including the Mississippi River Basin, Atlantic Coastal Plains, Gulf Coast Plains, Appalachian Mountains, Florida Peninsula, and in the western Coast Range, whereas it is almost nonexistent in the central plain. In China, *L. styraciflua* is distributed in humid climate regions, whereas it is not found in arid regions. Overall, these regions belong to temperate and subtropical climates. Based on these EI values of known distribution points, the cutoff value between the marginally favorable and favorable habitats was set at 15, and the cutoff value between the marginally favorable and favorable habitats was set at 30. The EI values were grouped into four classes: unfavorable habitat (0), marginally favorable habitat (0–15), favorable habitat (15–30), and highly favorable habitat (>30).

### 2.5. Using RF to Project the Potential Distribution of A. suncei

#### 2.5.1. Modeling Process

In this study, the RF model from the BioMod2 package in R was employed to simulate the potential distribution of *A. suncei* [44,45]. To minimize sampling bias due to occurrence records from various sources, 80 pseudo-presence points were randomly generated [17]. The model was then trained and validated using 10-fold cross-validation. Of the 10 subsets, a single subset was retained as the validation data for testing the model. The remaining subsets were used as training data. For each subset, 70% of the occurrence data were used to train the single model. The remaining data were used to test the predictive performance of the model. The model codes were executed 10 times for cross-validation, leading to improved accuracy, reduced errors, and more realistic predictions. The significance of effective environmental variables influencing the distribution of *A. suncei* was determined through the corresponding statistical analysis. This approach resulted in a more precise and realistic distribution map for *A. suncei*.

#### 2.5.2. Classification of Suitability for Potential Distribution

The output layers of the model results are raster layers with a probability from 0 to 1. The raster values represent the probability of the presence of *A. suncei* in that raster. The suitability level of *A. suncei* in each raster can be obtained by thresholding the probability layers [46]. The output layers were imported into ArcGIS, and the natural breaks (Jenks) method was used to classify the suitability levels of *A. suncei* [12]. The natural breaks (Jenks) method adheres to the principle of maximizing inter-group variance while minimizing intra-group variance to achieve accurate results [47]. In this study, a raster layer was divided into five area types in the parameter setting, which represent the suitability of unsuitable, very low, low, medium, and high habitats. Using this method, regions where the known occurrence coordinates are located were classified as high-suitability areas, which all belong to subtropical monsoon and monsoon humid climates. Almost all high-suitability areas of the globe were found in such climates. Areas with other climates were classified as low or unsuitable. Achieving this classification effect provided an accurate classification of the suitability for the potential distribution of *A. suncei.* As the thresholds of the natural breaks (Jenks) method may change according to the actual conditions of the layers in different periods, the specific threshold criteria were not marked, and only the suitability levels were shown in the map.

#### 2.5.3. Evaluation of Model Accuracy

The AUC, TSS, and Kappa values were used to evaluate the accuracy of the model under each climate scenario. AUC is the area under the receiver operating characteristic curve (ROC), with values ranging from 0 to 1 [48]. The Kappa coefficient is an index to evaluate the classification accuracy of the model based on the confusion matrix, with a range of −1 to 1 [49]. True skill statistics (TSS) is an improved test index derived from the kappa coefficient, which was used to evaluate the discrimination ability of the model by calculating the difference between the true positive rate and the false positive rate when the model predicted the positive and negative classes, with values ranging from 0 to 1 [50]. The closer the value of the above three indices was to 1, the higher the accuracy of the model.

### 2.6. Acquisition of Potential Distribution of A. suncei Concerning the Host L. styraciflua

We imported the prediction results of RF and CLIMEX into ArcGIS for processing. Firstly, the layer of CLIEMX was converted into a binary raster layer, in which the regions with EI = 0 had a value of 0, and the regions with EI > 0 had a value of 1. All the regions with a value of 1 were extracted to represent the regions where the host was present. Using the host presence region as a mask, the RF layer was extracted using the Extraction by Mask function; hence, the potential distribution area of *A. suncei* concerning the host was obtained [22].

## 3. Results

### 3.1. Potential Global Distribution of the Host L. styraciflua Predicted by CLIMEX

The accuracy of the final CLIMEX parameters depends on the recorded coordinates of *L. styraciflua* worldwide occurring within the predicted area ranges. Our result layer showed that 99.9% of the recorded coordinates were within the potential distribution predicted by CLIMEX, indicating that the final parameters of the model were reliable and highly accurate. The final parameters can, therefore, be used to predict, with high confidence, the potential distribution of *L. styraciflua.*

**Figure 3:**
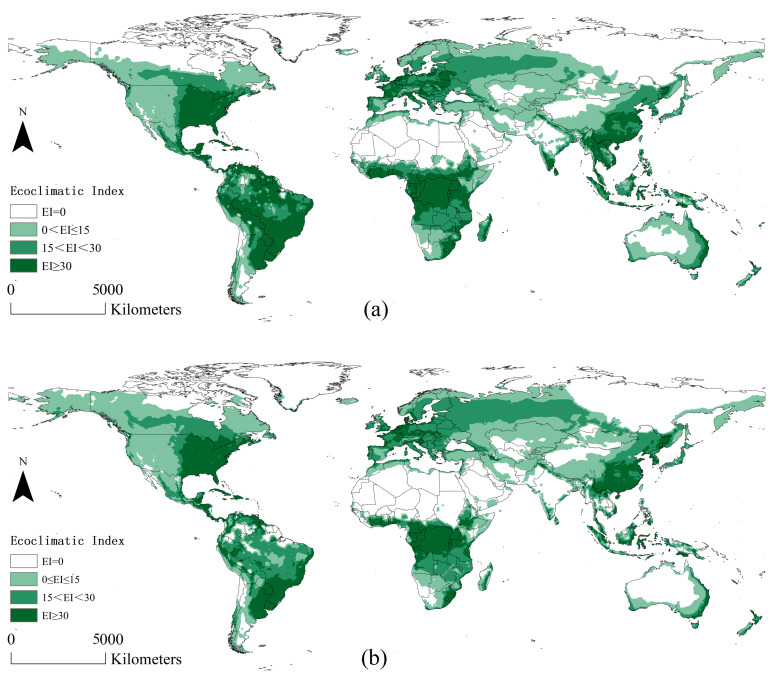
Potential global distribution of *L. styraciflua* predicted by CLEMEX; (**a**) distribution under current climate scenarios; (**b**) distribution under A1B climate scenarios in 2050.

**Figure 4:**
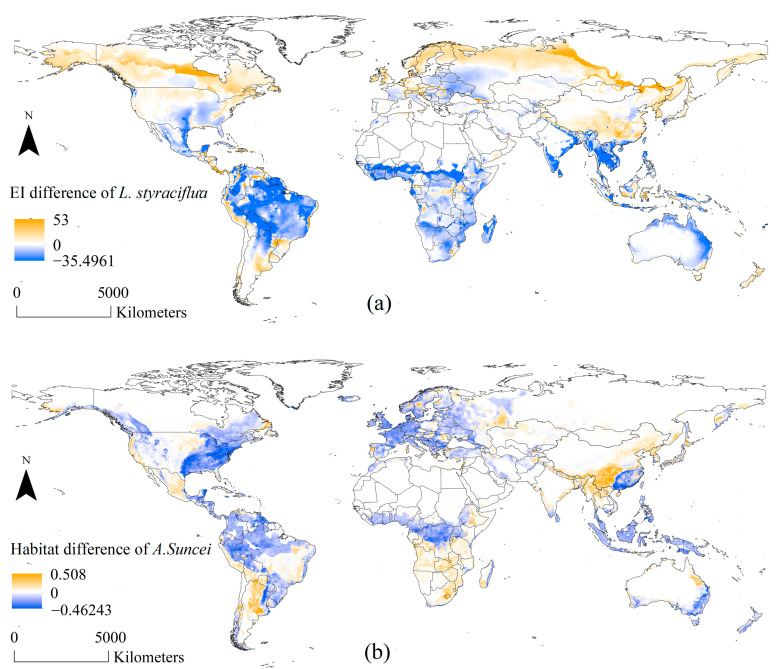
Habitat suitability difference from current to future climate scenarios: (**a**) habitat suitability difference in *L. styraciflua*; (**b**) habitat suitability difference in *A. suncei* (the value in each raster is calculated by the future suitability value minus the current suitability value).

Currently, *L. styraciflua* exhibits a broad, suitable range globally. The areas of EI ≥ 30 are concentrated in Eastern USA, Central America, central–southern South America, Eastern China, Southern India, Southeast Asia, Central Europe, Central Africa, and Eastern Australia (Figure 3). Figure 4a shows the difference in potential habitats of *L. styraciflua* from the present to the future. The yellow and blue areas represent the increased and decreased potential habitats, respectively. Under future climate conditions, the suitable areas of *L. styraciflua* in the Northern Hemisphere showed a clear tendency to migrate northward, expanding towards Northwest Russia, Northern Europe, Northern Canada, and Central China (Figure 4a). Areas suitable for *L. styraciflua* around the equator decreased. In the Southern Hemisphere, an overall trend of shrinking was observed for suitable areas for *L. styraciflua*. Overall, the total suitable area for *L. styraciflua* remained almost unchanged, varying from 9734.3 × 10^4^ km^2^ to 9778.6 × 10^4^ km^2^.

### 3.2. Potential Global Distribution of A. suncei Predicted by RF

According to the model results under the current climate scenarios, the values of the model evaluation indicators AUC, Kappa, and TSS were calculated to be 0.978, 0.934, and 0.900, respectively, while under the future climate scenarios, the values were 0.957, 0.862, and 0.916, respectively, which demonstrated that our model had sufficient accuracy.

**Figure 5:**
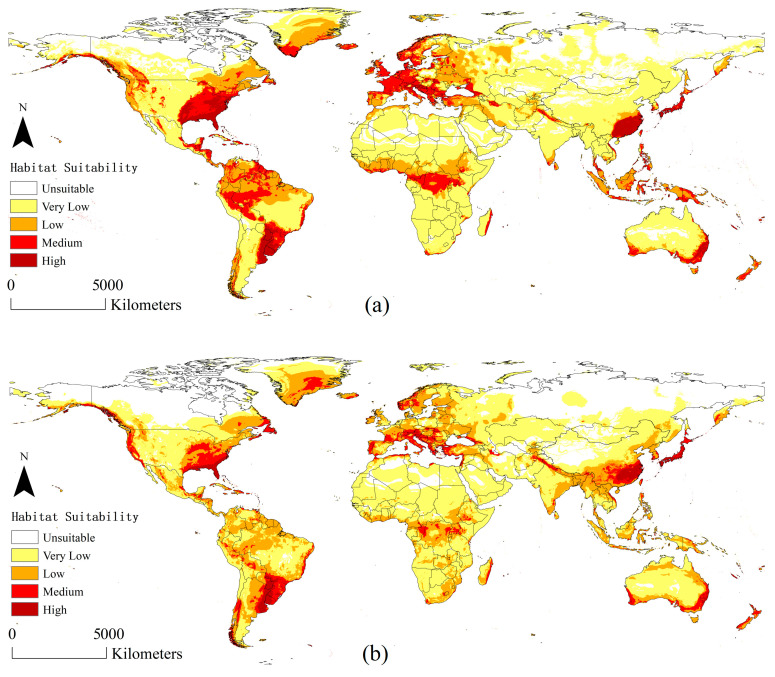
Potential global distribution of *A. suncei* predicted by Random Forests: (**a**) distribution under current climate scenario; (**b**) distribution under an RCP6.0 climate scenario in 2050.

Globally, under both current and future climate scenarios, the areas with high suitability are centered in Southern China, South Hokkaido in Japan, Southern USA, the La Plata Plain in South America, Southeastern Australia, and the Northern Mediterranean (Figure 5). The suitability levels gradually decreased in the surrounding areas centered on these areas with high suitability. Many areas with medium and low suitability have been detected near the equator.

In China, the low-suitability areas in Southwest and Northeast China have significantly increased. The medium-suitable areas also extend to Southwest and Central China. In Northern China, an increasing trend of low-suitability areas has been observed, extending along coastal provinces and cities towards higher latitudes, reaching the border between China and Russia directly. The low-suitability areas in Southeast Asian countries and South Asia bordering China, including Vietnam, Laos, Myanmar, Bangladesh, India, and Nepal, are projected to increase.

**Figure 6:**
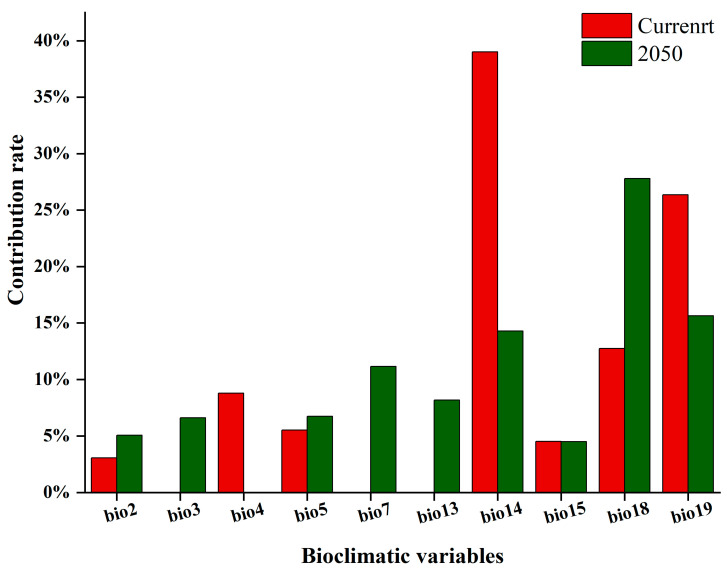
Contribution rates of important variables affecting the potential distribution of *A. suncei*.

The contribution of bioclimatic variables to the model results is shown in Figure 6. Under the current climate condition, the five main variables that contribute the most are the precipitation of the driest month (39.0%), precipitation of the coldest quarter (26.4%), precipitation of the warmest quarter (12.73%), temperature seasonality (8.8%), and maximum temperature of the warmest month (5.5%). Under the RCP6.0 climate scenario, the five main variables that contribute the most are precipitation of the warmest quarter (27.8%), precipitation of the coldest quarter (15.6%), precipitation of the driest month (14.3%), temperature annual range (11.1%), and precipitation of the wettest month (8.2%). In general, the total contribution rate of rainfall-related environmental variables surpasses that of temperature-related variables among the five key environmental variables. This suggests that *A. suncei* is more sensitive to precipitation than to temperature, providing valuable insights into how *A. suncei* may respond to climate change.

### 3.3. Global Potential Distribution of A. suncei Concerning the Host L. styraciflua

**Figure 7:**
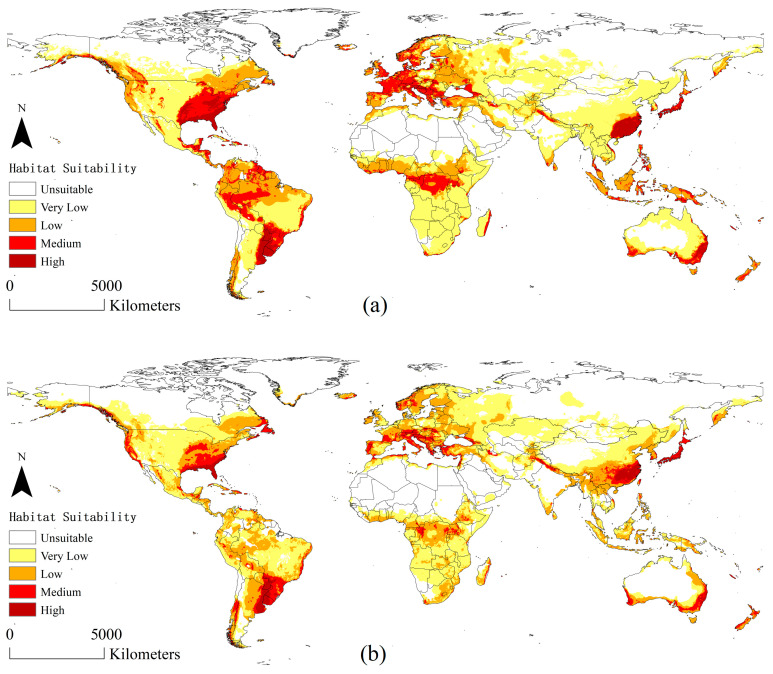
Potential global distribution of *A. suncei* concerning the host *L. styraciflua*: (**a**) distribution under current climate scenarios; (**b**) distribution under future climate scenarios in 2050.

Figure 7 shows the global distribution area of *A. suncei* after extraction using the suitable region of *L. styraciflua* as a host mask. The extraction results showed that almost all the areas with high or medium suitability of *A. suncei* were retained (Figure 7).

Figure 4b illustrates the difference in *A. suncei*-suitable areas from the current climate to the future using calculations based on Figure 7. The yellow and blue colors represent the increase and decrease in suitability in this region, respectively. As a whole, most of the current areas with high suitability will tend to decrease in suitability in the future. In Asia, the suitable areas in Southern China tend to shift towards Southwest, Central, and Northeast China. In North America, the most suitable areas in the USA tend to decrease in suitability. In South America, suitability significantly increases on the western side of the La Plata Plain and decreases in most areas. In Africa, suitable areas tend to migrate southward. In Europe, suitability decreases overall. In Oceania, Australia has a small tendency for suitable areas to migrate northward.

**Figure 8:**
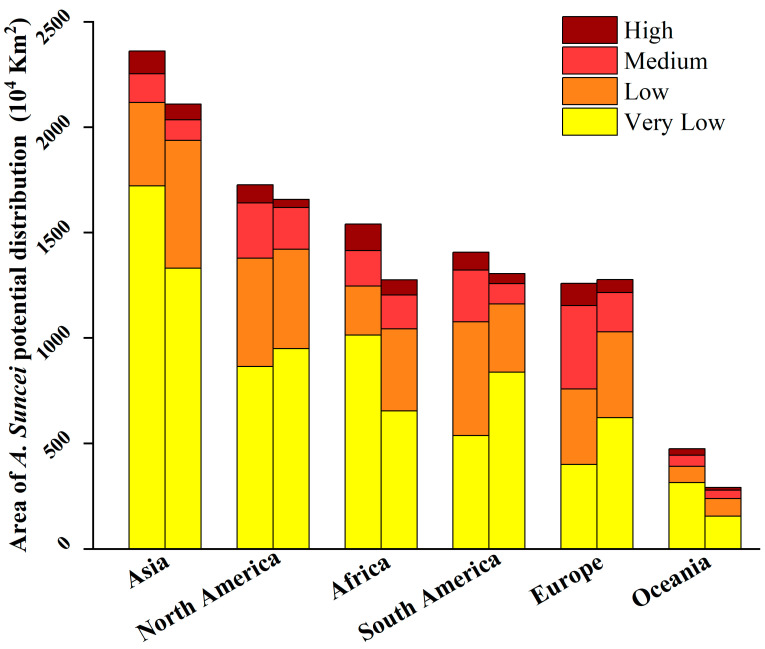
The area of *A. suncei* habitats of each suitability level in each continent under current climate scenarios (**left** column) and under the RCP6.0 climate scenario in 2050 (**right** column).

Figure 8 shows the current (left column) and future (right column) areas of each suitability level for each continent of *A. suncei* calculated based on Figure 6. Within each suitability level, very low suitability occupies most areas. The higher the suitability, the smaller the areas. As a whole, the total current and future suitable areas of the world are 8764.93 × 10^4^ km^2^ and 7914.02 × 10^4^ km^2^, respectively. The total suitable area will be reduced by 9.71% in the future.

## 4. Discussion

The climatically suitable areas of *A. suncei* are mainly distributed in Southern China, South Hokkaido in Japan, Southern USA, the La Plata Plain in South America, southeastern Australia, and the northern Mediterranean, which are located in subtropical monsoon and monsoonal humid climates or Mediterranean climate zones. The change is evident in several areas under the future climate scenario. The high- and medium-suitable areas in the Southeastern USA (inland), Central Europe, the La Plata Plain in South America, the Congo Basin in central Africa, and southeastern Australia show significant decreases. In China, signs of changes in suitable areas provide important insights into the survival and spread of this pest. The low-suitable areas in Southwest and Northeast China show significant increases. The medium-suitable area also extends to Southwest and Central China. In Northern China, an increasing trend of low-suitable areas is also observed, extending along coastal provinces and cities towards higher latitudes, reaching the border between China and Russia directly. Low-suitable areas in Southeast Asian countries and South Asia bordering China, including Vietnam, Laos, Myanmar, Bangladesh, India, and Nepal, are projected to increase. Previously, these areas had always been very low-suitable areas. Although the high-suitable areas in southeastern China show a decrease, the high-suitable areas in eastern China where the pest currently occurs remain unchanged. Regardless of current or future climate scenarios, eastern China will be the center of the occurrence and spread of this pest. According to this analysis, the pest may possibly spread in a direction centered in Eastern China, gradually spreading to Central, Southwestern, and Northeastern China, then Southeast and South Asia. The spread of this pest to other countries via the sea cannot be ignored. *A. suncei* can be spread over long distances via the human transportation of infected hosts [12]. Shanghai and Suzhou, where *A. suncei* occurs at the forefront of China’s international trade transactions, where the booming seaborne trade increases the risk of this beetle’s global spread. Susaeta et al. projected that once *A. suncei* spread into the USA, it would cause an economic loss of USD 152 million [13]. In conclusion, strict quarantine is a top priority to prevent the continued expansion of *A. suncei.*

Our results show that precipitation variables play a dominant role in the potential distribution of *A. suncei*. Whether under current or future climate scenarios, the cumulative contribution of precipitation variables exceeds 70%. The cumulative contribution rates of the top three variables, including precipitation of the driest month, precipitation of the coldest quarter, and precipitation of the warmest quarter, exceeded 50%. Precipitation is the key environmental variable that limits the potential distribution of *A. suncei*. The climatically suitable regions of *A. suncei* are distributed in subtropical monsoon and monsoonal humid climates or Mediterranean climate zones. Many studies have demonstrated that precipitation patterns (such as the uneven distribution of seasonal precipitation) are a key factor limiting the geographic distribution of numerous species located in these climate zones [51,52,53]. Based on the selected variable results, we believe that seasonal precipitation may limit the survival and dispersal ability of *A. suncei*. According to Wainwright et al., precipitation could affect the success rate of *A. suncei* population dispersal, thereby affecting its suitability [54]. During population dispersal, precipitation weakens its flight capabilities and reduces its likelihood of searching for a new host, hence reducing dispersal success [55]. *L. styraciflua* is sensitive to humidity. Based on Kriticos et al.’s reports, precipitation could affect host suitability, thereby influencing insect suitability [19]. Therefore, the change in precipitation pattern may be a key indicator to determine the successful invasion and colonization of *A. suncei*, which requires further exploration in future studies.

Although *A. suncei* currently feeds on *L. styraciflua* in its original habitat, this does not mean that its host is *L. styraciflua* alone. *A. suncei* initially fed on the Chinese native tree species *Liquidambar formosana*. *A. suncei* gradually specialized in feeding on *L. styraciflua* with its continuous promotion in China [6]. Therefore, *A. suncei* could also feed on tree species that are closely genetically related to *L. styraciflua*. If the beetle invades an area without its host trees, it might adapt by utilizing other host species to thrive in the new environment [56]. Consequently, if *A. suncei* spreads globally and adapts to more hosts, its actual distribution range may be larger than that simulated in our model. However, such results also have certain limitations. Other environmental factors such as human activities, insect–host relationships, host availability, and enemies are ignored, which may become important factors that affect prediction accuracy [57,58,59]. For instance, changes in the phenology of pests and their hosts due to climate change may lead to mismatches in their phenological cycles, potentially hindering pest survival by limiting the availability of nutrients necessary for their development [60]. Thus, it is essential to consider additional factors to improve our findings. The CLIMEX model has its limitations as well. Biological parameters for species vary across regions, yet many researchers apply uniform parameters when estimating global potential distributions, which can compromise model accuracy [61]. Furthermore, the physiological parameters of species can shift with climate change in different areas [62]. Hence, the spatial and temporal variations in these parameters are crucial to accurately describe species responses to changes in distribution areas and climate and enhance projection accuracy. Therefore, we need to continuously strengthen and improve these aspects in future studies.

In future climate conditions, global precipitation patterns will undergo significant changes [63]. Wet areas are known to become wetter, while dry areas become drier. Therefore, the results of these climate change projections are bound to affect the growth and development of pests and hosts. The shift in suitability due to climate change differs across regions. These results may assist us in pinpointing areas that are particularly sensitive to local climate change and assessing how suitability is affected under various climate change scenarios. Our projected results provide important information about the current and future potential distribution of *A. suncei* worldwide, which could be used as a valuable reference for identifying areas currently susceptible to and susceptible in the future to potential invasion by this pest. Particularly, with the increase in global trade, quarantine efforts should be strengthened in projected high and medium regions to prevent pest spread via the transportation of seedlings and other host trees. Long-term fixed-point monitoring should be undertaken, particularly in areas with high suitability. The task of monitoring where the beetle occurs in original habitats is a top priority. Biosecurity authorities throughout extremely favorable regions should be alerted to the invasion threat posed by this pest. This projection provides useful information for instigating long-term management strategies directed at reducing the favorable impact of future climate change on this pest.

## 5. Conclusions

This study predicted the global distributions of *L. styraciflua* and *A. suncei* under current and future climate scenarios using CLIMEX and Random Forests, respectively, and extracted the suitable distribution areas of *A. suncei* using the suitable areas of *L. styraciflua* as a mask to achieve the effect of predicting the distribution of *A. suncei* concerning the host. The results show that the high-suitability areas for *A. suncei* are primarily concentrated in Southern China, Japan South Hokkaido, the Southern United States, the La Plata Plain in South America, Southeastern Australia, and the Northern Mediterranean, totaling approximately 540.74 × 10^4^ km^2^. Seasonal rainfall plays an important role in environmental factors affecting the potential distribution of *A. suncei.* Under future climate conditions, most of the areas with high suitability will remain unchanged, suitability in many areas will change to varying degrees, and the total suitable area is projected to decrease by 9.71%. Based on these findings, we inferred the possible propagation direction of *A. suncei* in the future. Therefore, our research can provide guidance for the quarantine, prevention, and control of *A. suncei*.

## Figures and Tables

**Table 1:** CLIMEX parameter values for *L. styraciflua.*

CLIMEX Parameter	Final Parameter Value
Temperature requirements	
DV0—Lower temperature threshold (°C)	10
DV1—Lower optimum temperature (°C)	16
DV2—Upper optimum temperature (°C)	32
DV3—Upper temperature threshold (°C)	38
Soil moisture	
SM0—Lower soil moisture threshold	0.1
SM1—Lower optimal soil moisture	0.5
SM2—Upper optimal soil moisture	0.75
SM3—Upper soil moisture threshold	1.5
Cold stress	
TTCS—Cold stress temperature threshold (°C)	−28.9
THCS—Cold stress temperature rate (week^−1^)	0.9
Heat stress	
TTHS—Heat stress temperature threshold (°C)	40
THHS—Heat stress temperature rate (week^−1^)	0.2
Dry stress	
SMDS—Dry stress threshold	0.1
HDS—Dry stress rate (week^−1^)	0.01
Wet stress	
SMWS—Wet stress threshold	1.8
HWS—Wet stress rate (week^−1^)	0.1
Hot–wet stress	
TTHW—Hot–wet temperature threshold (°C)	28
MTHW—Hot–wet moisture threshold	2.8
PHW—Stress accumulation rate (week^−1^)	0.05

## Data Availability

The raw data supporting the conclusions of this article will be made available by the authors on request.

## Supplementary Materials

The following supporting information can be downloaded at https://www.mdpi.com/article/10.3390/insects15110897/s1. Table S1: Occurrence coordinates of *A. suncei* in China. Table S2: Global presence coordinates of *L. styraciflua.*
